# Ultra-processed food consumption and nutritional frailty in older age

**DOI:** 10.1007/s11357-023-00753-1

**Published:** 2023-02-24

**Authors:** Roberta Zupo, Rossella Donghia, Fabio Castellana, Ilaria Bortone, Sara De Nucci, Annamaria Sila, Rossella Tatoli, Luisa Lampignano, Giancarlo Sborgia, Francesco Panza, Madia Lozupone, Giuseppe Colacicco, Maria Lisa Clodoveo, Rodolfo Sardone

**Affiliations:** 1 Unit of Data Sciences and Technology Innovation for Population Health, National Institute of Gastroenterology “Saverio de Bellis”, Research Hospital, Castellana Grotte, Bari, Italy; 2 Institute of Clinical Physiology, National Research Council (IFC-CNR), Pisa, Italy; 3 Department of Translational Biomedicine and Neuroscience, University of Bari Aldo Moro, Bari, Italy; 4 Department of Translational Biomedicine and Neuroscience (DiBrain), University of Bari Aldo Moro, Bari, Italy; 5 Department of Interdisciplinary Medicine, University of Bari Aldo Moro, Bari, Italy; 6 Local Healthcare Authority of Taranto, Taranto, Italy

**Keywords:** Ultra-processed foods, Food intake, Food processing, Dietary habits, Older adults, Nutritional frailty

## Abstract

Frailty is a multidisciplinary public health issue and nutrition is key concern. Given the scientific consistency about inflammation as shared pathway to poor nutrition and frailty, food processing seems a suitable target to gain evidence in frailty prevention nutrition settings. This study aimed to assess diet in relation to nutritional frailty using the NOVA classification. Browsing the dataset of the Salus in Apulia, 2185 older adults were found to have completed the nutritional assessment, providing eligible data for this study goal. A validated construct, based on the co-presence of physical frailty by CHS criteria plus nutritional imbalance, was applied to characterize nutritional frailty phenotypes. Using the NOVA classification, daily food and beverage intakes from an 85-item self-administered FFQ were assigned to three categories, and effect sizes were tested among groups according to nutritional frailty status (presence/absence). Raw and adjusted logistic regression models were run to assess associations between NOVA food categories by quintiles of daily exposure (very-low, low, mild, moderate, high) and nutritional frailty. Nutritional frailty prevalence was 27%, being more frequent in males. Eating more unprocessed or minimally processed foods was inversely related to nutritional frailty, even after adjustment (OR: 0.10, 95%CI 0.07–0.16), showing a downward ORs behavior toward lower consumption quintiles. Listing in the quintile of moderate consumption of processed foods meant a nearly 50% increase in nutritional frailty probability (OR: 1.46, 95%CI 1.03–2.06), while the probability was double for the highest quintile against the lowest (OR: 3.22, 95%CI 2.27–4.58). A growing probability of nutritional frailty was found for increasing consumption of ultra-processed foods, but significance was lacking. The contribution of food processing to poor nutrition needs to be considered when promoting a better understanding of effective nutritional screening in aging. Therefore, food processing should be accounted for when composing diet guidelines for the older population within the framework of multidisciplinary efforts to ease the frailty healthcare burden.

**Supplementary information:**

The online version contains supplementary material available at 10.1007/s11357-023-00753-1.

## Introduction

As the burden of population aging grows [1], a multidisciplinary research effort probing risk biopathways is required to advance preventive strategies against frailty, an aging-related multifactorial phantom epidemic featuring poor resistance to stressors and low-grade systemic inflammation [2].

Nutritional imbalance, one facet of concern in the etiopathogenesis of this aging syndromic state, has attracted increasing attention of late [3, 4]. Much of the biology surrounding the connection between nutrition and frailty lies in the pathophysiology of aging, accompanied by a physiologically decreasing appetite and hence diminishing food servings, resulting in unintentional progressive weight loss. Beyond an impaired ability to recognize sensations such as hunger and thirst, older people have impaired nutrient absorption and utilization [5]. They are less likely to eat protein foods due to poor appetite, difficulties in chewing, as well as mental, financial, social [6], and cultural limitations [7]. Consequently, the failure to balance protein intake with requirements causes a mismatch between muscle protein synthesis and degradation, hence a loss of skeletal muscle mass [8]. The threat of a nutritional imbalance is serious because it complicates the trajectories of worsening frailty [9], making malnourished, older frail subjects more likely to fall ill and develop multimorbidity, disability, and reduced survival [10]. Our previous findings on the aging population from Southern Italy indicated that the combination of physical frailty and nutritional imbalance (defined as high dietary sodium versus low dietary iron and potassium intake) doubles the risk of death in such subjects compared to those with either frailty or nutritional imbalance alone [10]. Similarly, Wei and colleagues argued that poor nutrition alone was not significantly associated with an increased prevalence or incidence of functional disability, poor quality of life, or mortality, whereas poor nutrition together with pre-frailty or frailty was consistently associated with a substantial increase in the prevalence and incidence of diminishing functional outcomes and mortality [11].

Importantly, in recent years, inflammation has been associated with both physical frailty and poor nutrition as a shared trait. Assessing the role of pro-inflammatory foods in the diets of these individuals may be useful in gaining a better understanding of the biological mechanisms underlying nutritional frailty [12]. In this context, a large body of evidence has pointed to processed and ultra-processed foods (UPFs) as negative contributors to inflammation [13]. Indeed, it is acknowledged that diet is a modulator of inflammation, and a too-poor diet may weigh on the state of health. Still, there is limited research in epidemiology investigating the association between food processing and frailty status in the elderly population; by contrast, there is scientific consistency in the claim that diets with higher amounts of unprocessed foods and antioxidants are associated with lower oxidative stress and reduced levels of fluid inflammatory biomarkers [12].

The purpose of this study is to evaluate the association between different levels of UPFs and the likelihood of nutritional frailty using cross-sectional data obtained from older individuals from the Salus Study in Apulia. As a valuable tool for nutrition and public health research, policy and action, the NOVA system is being chosen and will be employed in this study as a globally recognized system that classifies foods into categories based on the extent of processing they have undergone.

## Material and methods

### Study population

**Fig. 1:**
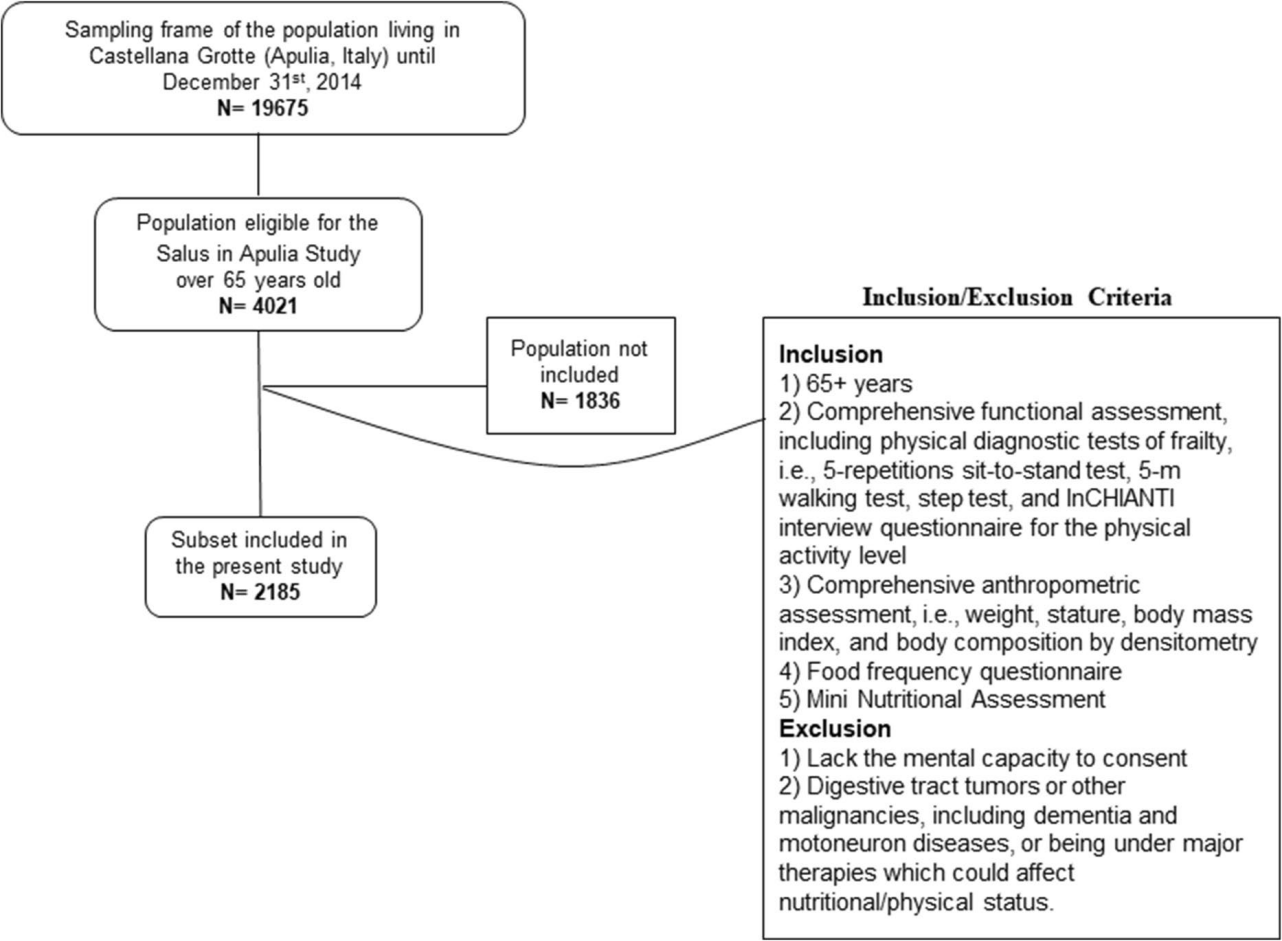
Informative flowchart

Participants of the present study were recruited from the electoral rolls of Castellana Grotte (Bari, Apulia, Southern Italy). All participants were part of the “Salus in Apulia Study,” which was undertaken at the National Institute of Gastroenterology IRCCS “S. De Bellis” Research Hospital and supported by the Italian Ministry of Health and the Apulia Regional Government. This is an ongoing longitudinal population-based study, activated in 2014, of a representative population of residents in Castellana Grotte (Apulia, southern Italy) who were 65 years of age or older at the time of initial recruitment. While the minimum age of 65 was required for enrollment in the Salus, conversely, the exclusion criteria were lack of mental capacity to express consent, having digestive tract tumors or other malignancies, including dementia and motoneuron diseases, or being under major therapies, which could affect nutritional/physical status. The study design and data collection method are detailed elsewhere [14, 15]. Briefly, the entire sampling frame consisted of the 4021 elderly residents in the health registry of the Apulia Region as of December 31, 2014. The study was born as multidisciplinary, including the assessments of the cognitive, sensory, physical, and nutritional domains, as illustrated in some of our previous works [16], and aimed to search for new biological and phenotypic determinants to predict and prevent risky trajectories of aging. Specifically, the data used for the present study came from a subset of the Salus, which included 2185 elders who had undergone all the examinations required for the purposes of this study. An informative flowchart is shown in Fig. 1. The IRB approved the study of the lead institution, the National Institute of Gastroenterology and Research Hospital “Saverio de Bellis,” and all subjects completed informed consent forms before their evaluation. The study met the principles of the Helsinki Declaration and adhered to the “Standards for Reporting Diagnostic Accuracy Studies” (STARD) guidelines (http://www.stard-statement.org/) and the “Strengthening the Reporting of Observational Studies in Epidemiology” (STROBE) guidelines. A STROBE checklist for cross-sectional studies is provided as Supplementary material.

### Clinical and laboratory examination

Education was defined by years of schooling. Smoking status was assessed with the single categorical question “Are you a current smoker?” (yes/no). A blood sample was collected in the morning after overnight fasting to measure the levels of glycated hemoglobin (HbA1c), total cholesterol, high-density lipoprotein (HDL) cholesterol, and low-density lipoprotein (LDL) cholesterol and triglycerides using standard automated enzymatic colorimetric methods (AutoMate 2550, Beckmann Coulter, Brea, CA, USA) under strict quality control. LDL cholesterol was calculated using the Friedewald equation. Plasma glucose was determined using the glucose oxidase method (Sclavus, Siena, Italy). Blood cell count was determined by a Coulter Hematology analyzer (Beckman–Coulter, Brea, CA). The clinical evaluation included extemporaneous ambulatory systolic blood pressure (SBP) and diastolic blood pressure (DBP) measurements, determined in a sitting position after at least a 10-min rest, at least three different times, using the OMRON M6 automatic blood pressure monitor. Serum high-sensitivity C-reactive protein (CRP) was assayed using a latex particle-enhanced immunoturbidimetric assay (Kamiya Biomedical Company, Seattle, WA) (reference range: 0–5.5 mg/L; interassay coefficient of variation: 4.5%). Serum interleukin (IL)-6 and tumor necrosis factor-alpha (TNF-α) were assayed using the quantitative sandwich enzyme technique of ELISA (QuantiKine High Sensitivity Kit, R&D Systems, Minneapolis, MN, and QuantiGlo immunoassay from R&D Systems, Minneapolis, MN). The interassay coefficient of variations was 11.7% for IL-6 and 13.0% for TNF-α. Inflammatory marker assays were analyzed at the same laboratory following strict quality control procedures. Multimorbidity status was defined as the co-presence of two or more chronic diseases as described in detail elsewhere [15].

### Anthropometric assessment

Two qualified nutritionists (RZ, SDN), trained for equivalent measuring performances, carried out the clinical procedures. All anthropometric measurements were taken with participants dressed in lightweight clothing and without shoes. Variables were all collected at the same time between 7:00 and 10:00 a.m., after overnight fasting. Height was measured to the nearest 0.5 cm using a wall-mounted stadiometer (Seca 711; Seca, Hamburg, Germany). Body weight was determined to the nearest 0.1 kg using a calibrated balance beam scale (Seca 711; Seca, Hamburg, Germany). BMI was calculated by dividing body weight (kg) by the square of height (m^2^) and classified according to World Health Organization criteria. Waist circumference (WC) was measured at the narrowest part of the abdomen or in the area between the tenth rib and the iliac crest (minimum circumference).

Bioelectrical impedance analysis (BIA) was performed using a single-frequency bioimpedance analyzer (BIA-101 analyzer, 50‐kHz frequency; Akern Bioresearch, Florence, Italy) to derive body composition estimates. The device was routinely checked with resistors and capacitors of known values. According to the European Society of Parenteral and Enteral Nutrition (ESPEN) guidelines [17, 18], all participants were examined in supine position with legs slightly apart, had abstained from eating, drinking, and exercise for 6 h and from drinking alcohol in the 24 h preceding the examination. Shoes and socks were removed, and contact areas were scrubbed with alcohol before electrode placement. Electrodes (BIATRODES Akern, Florence, Italy) placement was proximal to the phalangeal–metacarpal joint on the dorsal surface of the right hand and distal to the transverse arch on the superior surface of the right foot. Sensor electrodes were placed at the midpoint between the distal prominence of the radius and ulna of the right wrist, and between the medial and lateral malleoli of the right ankle. All measurements were supervised by a senior nutritionist (RZ) under strictly standardized conditions. Whole-body impedance vector components, resistance (R, Ω) and reactance (Xc, Ω), were derived and recorded when stable. The skeletal muscle index [SMI (kg/m^2^)] was obtained by dividing absolute muscle mass by squared height. Using the cutoff values indicated by the European Working Group on Sarcopenia in Older People [19], low muscle mass was classified as SMI less than 8.87 and 6.42 kg/m2 in men and women, respectively.

### Dietary intake and food classification according to NOVA

Diet was assessed with a self-administered Food Frequency Questionnaire (FFQ) to investigate dietary habits over the previous year. The semi-quantitative FFQ was structured in eleven sections that partly mirror the sequence of food intake during the day and include foods of similar characteristics: grains, meat, fish, milk and dairy products, vegetables, legumes, fruits, miscellaneous foods, water, and alcoholic beverages, olive oil and other edible fats, coffee/sugar, and salt. In a further step, the FFQ was validated against dietary records, and the results were reviewed to make any necessary modifications to the questionnaire [20]. The final questionnaire included 85 foods that best reflect the regional diet, along with some questions about the use of edible fats. One food group (cooking edible fats, the 19th of the list) could not be quantified and was not used in the present study [[21]. For the other food groups, daily intake was estimated. Supplementary Table 1 (STable 1) shows the concordance of the single foods in the questionnaire and the food grouping according to NOVA. We grouped all foods and beverages reported in the FFQ into three sets according to the NOVA classification [22]: (i) unprocessed or minimally processed foods; (ii) processed foods; (iii) UPFs (see detailed list of the foods in STable 1). We calculated total intake as the average daily consumption (g/day) of each NOVA food group and subgroup. Food intake (g/day) was standardized to an energy intake of 2000 kcal/day for description and comparison purposes. Standardization was done by dividing the daily food intake into grams/day by kcal/day and multiplying by 2000. Likewise, daily protein (mg/day) and protein/energy ratio (mg/kcal) intake were also estimated. Also, for descriptive purposes, estimates of daily alcohol consumption were derived from Italian food composition tables [23]and, according to American and European standards on daily alcohol consumption, a threshold of 20 g/day in females and 30 g/day in males was used [14, 24].

### Physical activity and physical frailty assessment

The assessment of physical frailty status was performed using a slightly modified version of the Fried operational definition [25], which requires the presence of three or more of the following criteria or components: weight loss, exhaustion, low levels of physical activity, weakness, and slowness. The 5-repetitions sit-to-stand test assesses the time it takes a patient to rise five times from a seated position without using their arms. It was employed as a surrogate measure of weakness, with a diagnostic threshold of > 15 s [26]. The Mini Nutritional Assessment, which gives weight loss and nutritional intake information, was used to evaluate nutritional status, with a score cutoff of 23.5 [27]. A 5-m walking test graded gait speed as slow if the recorded time was greater than or equal to the cutoff value of 0.6 m/s for slow gait speed. An interviewer administered a questionnaire to evaluate physical activity [28]. In particular, people were asked to choose from six response categories (ranging from 0 to 5) that include time, frequency, and intensity of physical activity to reflect their average level of physical activity over the last year. Based on the findings of a recent study on a subgroup of our sample that examined the association between activity energy expenditure derived from wrist-worn accelerometers and intensity of self-reported physical activity (InCHIANTI structured interview questionnaire) [29], we further dichotomized the variable using the cutoff value < 2. Using a modified version of the Berg stool-stepping task, the step test was applied as a measure of exhaustion [30]. The whole sample was assigned to two groups based on the number of physical frailty components. Subjects who met ≥ 3 criteria were included in the frailty group; all the others were classified in the non-frailty group.

### Nutritional imbalance and nutritional frailty assessment

Nutritional frailty was defined as the co-presence of physical frailty and a nutritional imbalance [10]]. The latter was assessed by the presence of two or more of the following five items: BMI < 21 kg/m^2^, SMI ≤ 8.87 for men or ≤ 6.42 for women, ≥ 2.3 g/day of dietary sodium, < 3.35 g/day of dietary potassium, and < 9.9 g/day of dietary iron. BMI and SMI cutoff values were set according to validated values [31]. As detailed in our previous study, the diet items were selected after stepwise skimming of a backward random survival forest (RSF) algorithm from the cluster of dietary variables studied. The cutoff value for nutritional imbalance had been evaluated based on the receiver operating characteristic (ROC) curve for death.

### Multimorbidity and non‑communicable diseases

Multimorbidity was defined as the co-presence at the baseline examination of two or more major non-communicable diseases, i.e., diabetes mellitus, hypertension, peripheral age-related hearing loss (ARHL), vision loss, cognitive impairment (as defined by the Mini-Mental State Examination, MMSE) [32], asthma, chronic obstructive pulmonary disease (COPD), and late-life depression (LLD), as described elsewhere [15, 16, 33]

### Statistical analysis

The whole sample was divided according to nutritional frailty phenotype (presence/absence) to compare differences in NOVA food intake. Normal distributions of the quantitative variables were tested using the Kolmogorov–Smirnov test. Due to the normal distribution of the variables, data are reported as mean (M) ± standard deviation (SD) for continuous measures and frequency and percentages (%) for all categorical variables.

The effect sizes (ES) of the NOVA food categories across the 2 groups of nutritional frailty conditions (presence/absence) were presented as standardized mean differences (Hedges’ g) and 95% confidence interval [34]. To evaluate the association of nutritional frailty with the three categories of NOVA foods divided into quintiles of cumulative daily consumption (very low, low, mild, moderate, and high), a logistic regression model, unadjusted, and adjusted (for age, gender, and education in model 2, and also for IL-6, CRP TNF-alpha, alcohol consumption, proteins/energy ratio, total daily energy intake, and multimorbidity in a further model 3) was built and the estimators were reported as odds ratios (ORs) and 95% confidence intervals. A sensitivity analysis for each model was also performed and provided as Supplementary Table 3 (STable 3). Sensitivity analysis ascertains how different values of an independent variable affect a particular dependent variable under a given set of assumptions and how various sources of uncertainty in a mathematical model contribute to the overall uncertainty of the model. The following measures were estimated, as follows. The C-statistic as a measure of the goodness of fit for binary outcomes in a logistic regression model; the AIC as a measure of the goodness of fit of any estimated statistical model; the BIC as a type of model selection among a class of parametric models with a different number of parameters; and the R-square on a similar scale, ranging from 0 to 1, with higher values indicating better model fit.

The methodological approach design and statistical analyses were managed by a senior epidemiologist (RS) and a biostatistician (RD) using StataCorp. 2021. Stata statistical software: Release 17. College Station, TX: StataCorp LLC.

## Results

The examined population (*N* = 2185) had a mean age of 73.56 ± 6.30 years and was slightly dominated by males (50.49%, *N* = 1103). Nutritional frailty prevalence was 27.64% (*N* = 604), being more frequent in males (60.69%, *N* = 352 out of 604).

Table 1 summarizes the main differences in socio-demographic, clinical, and diet variables, as expressed by cumulative daily NOVA food intake (g/day) by consumption quintiles (very low, low, mild, moderate, and high) of the whole sample subdivided according to the nutritional frailty condition (presence/absence). Functional analysis showed poorer SMI values, and biochemistry demonstrated significantly lower mean platelet values in the nutritional frailty group (ES 0.30, 95%CI 0.21 to 0.40 and ES 0.10, 95%CI 0.001 to 0.19, respectively).

**Table 1:** Socio-demographic, clinical, and dietary (g/day) variables according to the NOVA classification by quintiles (very low, low, mild, moderate, and high) in the whole sample by nutritional frailty phenotype (presence/absence)

Parameters^^^	Nutritional Frailty			
	No (*n* = 1581)	Yes (*n* = 604)		Effect size^ψ^ (95% CI)
Age (years)	73.48 ± 6.24	73.73 ± 6.43		− 0.04 (− 0.13 to 0.06)
Gender (M) (%)	622 (46.11)	352 (60.69)		0.14 (0.10 to 0.19)
Education (years)	6.87 ± 3.80	7.09 ± 3.88		− 0.06 (− 0.15 to 0.04)
Low physical activity (yes) (%)	1122 (83.17)	477 (82.24)		− 0.01 (− 0.05 to 0.03)
Skeletal muscle index (SMI)	8.58 ± 1.60	8.08 ± 1.65		0.30 (0.21 to 0.40)
Weight loss (yes) (%)	97 (7.19)	32 (5.52)		− 0.02 (− 0.04 to 0.01)
Exhaustion	159 (11.79)	69 (11.90)		0.001 (− 0.03 to 0.03)
Weakness	429 (31.80)	177 (30.52)		− 0.01 (− 0.06 to 0.03)
Slow gait	361 (26.76)	133 (22.93)		− 0.04 (− 0.08 to 0.003)
Multimorbidity (≥ 2) (%)	621 (46.03)	260 (44.83)		− 0.01 (− 0.06 to 0.04)
Proteins intake (mg/die)	74.80 ± 44.75	71.49 ± 32.24		0.08 (− 0.02 to 0.19)
Energy intake (standardized)	0.02 ± 1.03	− 0.03 ± 1.03		0.05 (− 0.06 to 0.15)
Proteins/energy ratio	0.04 ± 0.01	0.04 ± 0.01		0.08 (− 0.03 to 0.18)
Alcohol (yes) (%)	0.19 ± 0.40	0.17 ± 0.38		0.05 (− 0.05 to 0.16)
Biomarkers				
Total cholesterol (mg/dL)	184.23 ± 36.75	182.30 ± 38.32		0.05 (− 0.04 to 0.15)
HDL cholesterol (mg/dL)	48.84 ± 13.21	48.11 ± 12.58		0.05 (− 0.04 to 0.15)
LDL cholesterol (mg/dL)	112.65 ± 30.75	112.77 ± 32.22		− 0.004 (− 0.10 to 0.09)
Triglycerides (mg/dL)	107.22 ± 60.85	104.58 ± 61.95		0.04 (− 0.05 to 0.14)
Systolic blood pressure (mmHg)	132.86 ± 14.22	133.51 ± 15.10		− 0.04 (− 0.14 to 0.05)
Diastolic blood pressure (mmHg)	77.97 ± 7.91	78.36 ± 7.99		− 0.05 (− 0.15 to 0.05)
Hemoglobin (g/dL)	13.75 ± 1.51	13.84 ± 1.49		− 0.06 (− 0.16 to 0.04)
Red blood cells (10^6^/µL)	4.76 ± 0.52	4.87 ± 1.58		− 0.11 (− 0.21 to − 0.02)
Platelets (10^3^/µL)	224.52 ± 61.34	218.58 ± 58.38		0.10 (0.001 to 0.19)
White blood cells (10^3^/µL)	6.09 ± 1.61	6.21 ± 2.18		− 0.06 (− 0.16 to 0.03)
Vitamin D (nmol/L)	39.20 ± 17.60	38.69 ± 17.95		0.03 (− 0.07 to 0.12)
HbA1c (mmol/mol)	40.50 ± 10.53	40.51 ± 10.49		− 0.001 (− 0.10 to 0.10)
AST (U/L)	30.37 ± 25.82	33.44 ± 31.27		− 0.11 (− 0.23 to 0.02)
ALT (U/L)	25.15 ± 17.39	25.50 ± 24.44		− 0.02 (− 0.14 to 0.11)
IL-6 (pg/mL)	4.00 ± 6.93	3.79 ± 6.21		0.03 (− 0.06 to 0.13)
TNF-α (µg/mL)	2.70 ± 3.17	3.06 ± 4.57		− 0.10 (− 0.20 to − 0.001)
PCR (mg/dL)	0.58 ± 0.90	0.60 ± 0.76		− 0.03 (− 0.13 to 0.07)
Unprocessed and minimally processed foods				
Very low	990.05 ± 194.70	959.60 ± 231.41		0.14 (− 0.10 to 0.38)
Low	1430.59 ± 93.47	1418.81 ± 90.98		0.13 (− 0.11 to 0.36)
Mild	1744.50 ± 94.28	1751.56 ± 100.37		− 0.07 (− 0.31 to 0.16)
Moderate	2130.49 ± 130.14	2114.16 ± 140.68		0.12 (− 0.13 to 0.37)
High	3111.37 ± 707.79	2920.09 ± 622.60		0.27 (− 0.05 to 0.59)
Processed foods				
Very low	64.66 ± 20.15	60.62 ± 20.92		0.20 (− 0.05 to 0.44)
Low	114.87 ± 13.84	117.51 ± 13.94		− 0.19 (− 0.44 to 0.06)
Mild	160.58 ± 13.17	162.38 ± 13.80		− 0.13 (− 0.37 to 0.11)
Moderate	231.26 ± 35.19	245.72 ± 39.99		− 0.39 (− 0.62 to − 0.15)
High	402.17 ± 109.22	409.66 ± 96.34		− 0.07 (− 0.31 to 0.17)
Ultra-processed foods				
Very low	18.46 ± 7.83	18.12 ± 8.01		0.04 (− 0.19 to 0.28)
Low	41.38 ± 5.92	40.35 ± 5.07		0.18 (− 0.06 to 0.42)
Mild	62.42 ± 6.75	62.15 ± 6.21		0.04 (− 0.20 to 0.28)
Moderate	91.09 ± 10.34	91.37 ± 10.65		− 0.03 (− 0.26 to 0.21)
High	191.53 ± 159.23	211.16 ± 129.77		− 0.13 (− 0.37 to 0.10)

HDL, high-density lipoprotein; LDL, low-density lipoprotein; HbA1, glycated hemoglobin; AST, aspartate transaminase; ALT, alanine aminotransferase; IL-6, interleukin-6; TNF-α, tumor growth factor-α; PCR, C-reactive protein

^^^As mean and standard deviation for continuous variables, percentage (%) for categorical variables

^*^The distribution was divided into quintiles

^ψ^Hedges’ effect size; (95% C.I.), 95% confidence intervals

As regards unprocessed or minimally processed foods, nutritional frailty phenotypes fell much more within the lowest quintile of consumption, labeled very low (30.85%, *N* = 178 out of 604 showing a nutritional frailty phenotype) with a progressive down-trend in proportions when moving toward the quintiles of highest consumption (STable 2). In fact, the nutritional frailty phenotype accounted for 25.65% (*N* = 148), 20.8% (*N* = 120), 15.08% (*N* = 87), and 7.63% (*N* = 44) in the low, mild, moderate, and high consumption quintiles, respectively. When comparing groups, the ES showed significant differences in the proportions within the very low, low, moderate, and high quintile of consumption (ES: 0.18, 95%CI 0.14 to 0.23, ES: 0.09, 95%CI 0.05 to 0.14, ES: − 0.08, 95%CI − 0.12 to − 0.04, and ES: − 0.21, 95%CI − 0.25 to − 0.17, respectively). However, when looking at the cumulative daily consumption of NOVA food categories, as expressed by quintiles of exposure, there were no significant differences compared to the nutritionally balanced non-frail counterparts in terms of ES between groups.

As to processed foods, nutritional frailty phenotypes fell much more within the highest quintile of consumption (30.50%, *N* = 176 out of 604 showing a nutritional frailty phenotype), revealing significant differences in proportions in the between-group comparison (ES: 0.18, 95%CI 0.13 to 0.22). Statistically, differences in proportions were also evident within the very low and the low- quintiles of processed food consumption (ES: -0.06, 95%CI − 0.10 to − 0.02, and ES: − 0.09, 95%CI − 0.13 to − 0.05). When expressing daily consumption in grams, significant differences between groups were found only within the moderate consumption quintile (ES: − 0.39, 95%CI − 0.62 to − 0.15).

Lastly, we found no significant differences between groups by proportions when testing the UPF category. However, the highest percentage of nutritional frailty phenotypes fell into the high-consumption quintile (21.66%, *N* = 125 out of 604, showing a nutritional frailty phenotype). Furthermore, no statistical differences emerged between groups when analyzing grams of UPFs daily consumption. A sensitivity analysis for each model has been provided as Supplementary Table 3 (STable 3).

Each of the three NOVA food categories quintiles was then entered into logistic regression models adjusted for major confounders (age, gender, education) to assess the odds of nutritional frailty for every different quintile of daily intake of the three levels of UPFs according to the NOVA classification (Table 2). A higher consumption of unprocessed or minimally processed foods was inversely associated with nutritional frailty (OR 0.10, *SE* 0.02, 95%CI 0.07 to 0.16). When inflammatory variables such as CRP, IL-6, TNF-alpha, as well as multimorbidity, were included in the adjusted model, they appeared not to affect the direction of the association (OR of about 1), contrary to the total daily energy intake (OR 2.03, 95%CI 1.70–2.43); instead, the inclusion of covariates such as alcohol consumption and daily total protein/energy ratio affected the direction in a protective way (OR < 1). In the fully adjusted models, the protective effect of low consumption of UPFs declined in power (showing decreasing ORs) in line with the increasing consumption quintiles (ORs 0.82, 0.82, 0.89, 1.06, respectively for low, mild, moderate, and high UPF consumption). Similarly, higher ORs for nutritional frailty status resulted for the medium, moderate, and high consumption of processed foods categories (ORs 1.11, 1.46, and 3.22, respectively), with confidence intervals signaling significance for both the moderate and high consumption categories. Specifically, individuals with a moderate daily consumption of processed foods had an almost 50% higher probability of nutritional frailty than those with a very low consumption (OR: 1.46, 95%CI 1.03 to 2.06). In the highest consumption quintile, the situation was even worse, since adjusted models showed that the likelihood of progression to nutritional frailty was more than double that for subjects in the lowest consumption quintile of processed foods (OR: 3.22, 95%CI 2.27 to 4.58). Again, the inclusion of inflammatory variables such as CRP, IL-6, TNF-alpha, as well as multimorbidity, in the adjusted model did not influence the outcome (OR of about 1), while covariates such as alcohol consumption, protein/energy ratio, and total dietary energy moved the direction of association in a protective way. Lastly, the models on UPFs quintiles showed a higher OR with respect to the nutritional frailty condition for those individuals taking a high daily consumption compared to a very-low, and this feature was maintained in both the raw and fully adjusted models for age, sex, and schooling (ORs 1.06 and 1.01, respectively), although lacking statistical significance. Once again, the inclusion of inflammatory variables such as CRP, IL-6, TNF-alpha, as well as multimorbidity, in the adjusted model did not influence the outcome (OR of about 1), while covariates such as alcohol consumption, protein-to-energy ratio, and total dietary energy shifted the direction of the association in a protective direction. Logistic models showed some protection of the female gender on nutritional frailty (ORs 0.58, 0.69, and 0.62 across increasing food processing models); older age seemed to have a detrimental effect only in the middle level of consumption of processed foods (OR 1.02, 95%CI 1.00 to 1.04), while education lacked significance, likely due to a misdistribution resulting from widespread poor schooling in this whole population.

**Table 2:** Logistic regression model for nutritional frailty of food groups according to the NOVA classification by quintiles (very low, low, mild, moderate, and high)

Parameters^*^	Univariate model			Adjusted model 1			Adjusted model 2			Adjusted model 3		
	OR	SE (OR)	95% CI	OR	SE (OR)	95% CI	OR	SE (OR)	95% CI	OR	SE (OR)	95% CI
Unprocessed and minimally processed												
Very low (ref.)	–	–	–	–	–	–	–	–	–	–	–	–
Low	0.64	0.11	0.46 to 0.90	0.65	0.11	0.47 to 0.92	0.66	0.11	0.47 to 0.92	0.48	0.09	0.33 to 0.68
Mild	0.43	0.07	0.31 to 0.61	0.43	0.07	0.31 to 0.61	0.43	0.07	0.31 to 0.61	0.26	0.05	0.18 to 0.38
Moderate	0.26	0.05	0.18 to 0.37	0.27	0.05	0.19 to 0.38	0.27	0.05	0.19 to 0.38	0.12	0.03	0.08 to 0.19
High	0.11	0.02	0.07 to 0.16	0.10	0.02	0.07 to 0.16	0.10	0.02	0.07 to 0.16	0.02	0.01	0.01 to 0.04
Age	–	–	–	1.01	0.01	0.99 to 1.03	1.01	0.01	0.99 to 1.03	1.01	0.01	0.99 to 1.03
Sex (female)	–	–	–	0.58	0.07	0.46 to 0.72	0.58	0.07	0.46 to 0.73	0.54	0.07	0.42 to 0.71
Education	–	–	–	–	–	–	1.00	0.01	0.97 to 1.04	0.99	0.02	0.96 to 1.02
IL-6	–	–	–	–	–	–	–	–	–	0.98	0.01	0.96 to 1.00
TNF-α	–	–	–	–	–	–	–	–	–	1.03	0.02	0.99 to 1.06
CRP	–	–	–	–	–	–	–	–	–	1.03	0.07	0.89 to 1.19
Alcohol (yes)*	–	–	–	–	–	–	–	–	–	0.35	0.06	0.25 to 0.51
Proteins/energy ratio	–	–	–	–	–	–	–	–	–	1.28e-07	8.60e-07	2.45e-13 to 0.07
Energy intake (standardized)	–	–	–	–	–	–	–	–	–	2.03	0.19	1.70 to 2.43
Multimorbidity	–	–	–	–	–	–	–	–	–	0.85	0.09	0.45 to 1.63
Processed foods												
Very low (ref.)	–	–	–	–	–	–	–	–	–	–	–	–
Low	0.85	0.15	0.59 to 1.21	0.85	0.16	0.60 to 1.22	0.85	0.15	0.59 to 1.22	1.32	0.25	0.91 to 1.93
Mild	1.12	0.20	0.79 to 1.59	1.12	0.20	0.79 to 1.59	1.11	0.20	0.78 to 1.58	2.37	0.47	1.60 to 3.52
Moderate	1.53	0.27	1.08 to 2.15	1.47	0.26	1.04 to 2.07	1.46	0.26	1.03 to 2.06	4.56	1.00	2.96 to 7.03
High	3.37	0.59	2.38 to 4.76	3.23	0.58	2.28 to 4.59	3.22	0.58	2.27 to 4.58	26.79	7.94	14.99 to 47.89
Age	–	–	–	1.02	0.01	1.00 to 1.03	1.02	0.01	1.00 to 1.04	1.01	0.01	0.99 to 1.03
Sex (female)	–	–	–	0.68	0.08	0.55 to 0.86	0.69	0.08	0.55 to 0.87	0.54	0.07	0.42 to 0.69
Education	–	–	–	–	–	–	1.01	0.01	0.98 to 1.04	0.99	0.02	0.97 to 1.03
IL-6	–	–	–	–	–	–	–	–	–	0.99	0.10	0.97 to 1.00
TNF-α	–	–	–	–	–	–	–	–	–	1.02	0.01	0.99 to 1.05
CRP	–	–	–	–	–	–	–	–	–	0.99	0.07	0.86 to 1.13
Alcohol (yes)	–	–	–	–	–	–	–	–	–	0.79	0.13	0.56 to 1.10
Proteins/energy ratio	–	–	–	–	–	–	–	–	–	0.0001	0.00001	5.86e-11 to 2.17
Energy intake (standardized)	–	–	–	–	–	–	–	–	–	0.35	0.04	0.28 to 0.43
Multimorbidity	–	–	–	–	–	–	–	–	–	0.98	0.01	0.38 to 1.63
Ultra-processed food												
Very low (Ref.)	–	–	–	–	–	–	–	–	–	–	–	–
Low	0.84	0.14	0.60 to 1.17	0.82	0.14	0.58 to 1.15	0.82	0.14	0.58 to 1.15	0.87	0.15	0.61 to 1.22
Mild	0.82	0.14	0.58 to 1.15	0.82	0.14	0.59 to 1.16	0.82	0.14	0.58 to 1.15	0.89	0.16	0.63 to 1.27
Moderate	0.92	0.16	0.65 to 1.28	0.89	0.15	0.63 to 1.25	0.89	0.15	0.63 to 1.25	0.97	0.18	0.68 to 1.39
High	1.07	0.18	0.76 to 1.49	1.06	0.18	0.75 to 1.48	1.06	0.18	0.76 to 1.49	1.26	0.25	0.86 to 1.85
Age	–	–	–	1.01	0.01	0.99 to 1.03	1.01	0.01	0.99 to 1.03	1.01	0.01	0.99 to 1.03
Sex (female)	–	–	–	0.61	0.07	0.49 to 0.76	0.62	0.07	0.50 to 0.77	0.54	0.06	0.42 to 1.03
Education	–	–	–	–	–	–	1.01	0.01	0.98 to 1.04	1.00	0.01	0.97 to 1.03
IL-6	–	–	–	–	–	–	–	–	–	0.99	0.01	0.97 to 1.00
TNF-α	–	–	–	–	–	–	–	–	–	1.03	0.01	0.99 to 1.06
CRP	–	–	–	–	–	–	–	–	–	0.99	0.06	0.88 to 1.13
Alcohol (yes)	–	–	–	–	–	–	–	–	–	0.69	0.11	0.51 to 0.94
Proteins/energy ratio	–	–	–	–	–	–	–	–	–	0.001	0.001	2.11e-0.9 to 11.95
Energy intake (standardized)	–	–	–	–	–	–	–	–	–	0.88	0.06	0.77 to 1.01
Multimorbidity	–	–	–	–	–	–	–	–	–	0.98	0.05	0.97 to 1.03

Adjusted model 1: corrected for age and gender. Adjusted model 2: corrected for age, gender, and education. Adjusted model 3: corrected for age, gender, education, inflammatory cytokines, alcohol intake (yes), protein/energy ratio, energy intake (standardized), and multimorbidity

OR, odds ratio; SE (OR), standard error of OR; 95% CI, 95% confidence intervals

^*^Alcohol consumption: yes if > 50 g/day or 18,250 g/year (males), and > 25 g/day or 9125 g/year (females)

## Discussion

The present study aimed to contribute further evidence of the burden of food processing on poor nutrition, analyzing the aging population belonging to the Salus in Apulia study using the nutritional frailty phenotype as the outcome. The main finding was an increasing OR between quintiles of increasing processed and UPF consumption and an inverse pattern for unprocessed or minimally processed foods. In practical terms, the probability of nutritional frailty increased by nearly 50% for moderate daily consumption of processed foods and doubled the odds for high versus very low consumption; similarly, there was an increasing probability for higher consumption of UPF, although here statistical significance was lacking.

The scientific rationale driving our research effort was the consistent literature describing inflammation as a common feature of physical frailty and poor nutrition in older age [12], as well as the quantitative (e.g., amount of energy intake) and qualitative (e.g., nutrient quality, especially protein supply) importance of diet in the development of frailty [35]. Processed and ultra-processed foods are notoriously palatable, ready-to-eat, and highly energy-dense [36, 37]. Due to these qualities, there is a greater risk of overconsumption and hence, in the aging setting, of a possible contribution to increased daily energy intake. However, their consumption has been associated with worse dietary quality (less fiber, fruit, and vegetables) [38], and, according to preliminary longitudinal data, this contributes to the frailty incidence [39]. Our findings indicate that nutritional frailty phenotypes are more accustomed to consuming processed foods and UPFs than their counterparts. These foods are a good choice in terms of food security, ensuring immediate food availability (helpful in the case of disability), and nutritional safety [37]. However, they lack nutritional quality, being convenience foods or beverages mainly or entirely formulated using food-derived substances and additives, with little or no natural, unaltered foodstuffs, and thus are described as part of unhealthy dietary patterns linked to adverse health outcomes, such as overall mortality, cardiovascular disease, metabolic syndrome, physical and cognitive decline, cancer, and others [40].

Our findings on the likelihood of developing nutritional frailty were in a protective direction for consumers of unprocessed or minimally processed foods. The OR was smaller than one but showed an increasing tendency after adjustment for daily dietary energy load and a clear decreasing tendency for total protein/energy ratio. This finding aligns with a body of previous research, such as those from the scientists of the French Three-City Cohort [41], describing how higher protein—but not energy—intake was associated with a lower prevalence of frailty and physical decline. However, we know that a positive energy balance easily promotes weight gain and increased BMI; this point, from a late-life perspective, may be meant to protect against physical decline or undernutrition, but the concept clashes with the widespread condition of sarcopenic obesity, which instead goes in a worsening direction on overall health status. In this sense, it is useful to distinguish between the protein component and the energy load and to keep in mind that adequate caloric intake is necessary for optimal utilization of the energy load [42]. Then, the entry of alcohol into the adjusted model moved the OR downward, indicating some sort of protection for subjects with usual drinking patterns, that is, a finding previously observed in the same Salus population but also elsewhere in Mediterranean settings [10, 21, 43]. Here, there has been speculation that the high concentration of polyphenols in wine may confer some health benefits and, overall, increase survival rate [44].

Conversely, findings on the odds of developing nutritional frailty showed double the risk for high consumption of processed foods compared with very low. Here, because there is still little knowledge about nutritional frailty phenotypes, comparing the findings with other studies is not feasible. However, to date, a single research work has provided prospective data investigating UPFs consumption against incident physical frailty in a Spanish population. Sandoval-Insausti and colleagues found that participants with the highest consumption, in the top quintile of UPFs intake, had a threefold increased risk of incident frailty in 3.5 years, compared with those with the lowest consumption, even after adjustment for confounders [35]. Noteworthy is the fact that the logistic models were not modified, for any of the NOVA groups exposed, by the inclusion of inflammatory variables such as CRP, IL-6, and TNF-alpha. Probably, because the target population was aged and tended physiologically to have higher-than-normal levels of inflammation, as shown in the descriptive analyses for groups, the condition of physical decline did not have a significant and consistent impact on the model, and ultimately, inflammation values did not significantly shift the ORs in our analyses.

Some assumptions might be advanced from a nutritional perspective. Processed foods are convenient, affordable, and aggressively marketed, which encourages constant snacking and may isolate less processed foods from the diet, resulting in a deterioration in diet quality (lower intake of fruits, fiber, and vegetables) that contributes to frailty. Our findings are consistent with those of previous recent reports that high adherence to the Mediterranean diet, known to be low in UPFs, was negatively associated with the incidence of frailty [45, 46]. Possible mechanisms proving the protective role of the Mediterranean diet toward frailty are its positive effect on biomarkers of endothelial dysfunction, insulin resistance, oxidation, and inflammation, which are part of the biological pathway of frailty. Second, the Mediterranean diet is well-balanced in bromatology, providing sufficient micronutrient and protein intake, which is associated with lower frailty. Also, a Westernized dietary pattern of high consumption of refined bread products and processed meats has been correlated with an increased risk of frailty [47]. Moreover, since the nutritional imbalance algorithm features low potassium and high sodium, our findings are consistent with the reduced potassium-to-sodium ratio suggested by bromatological estimates of processed foods [48]. Indeed, processed and restaurant foods often carry a higher sodium content due to palatability or safety reasons, and therefore, this trend likely contributes to high sodium consumption [49]. In contrast, more than half of the ultra-processed products seem to have an insufficient potassium content, although potassium is well known to be an essential mineral that helps to regulate fluid balance, muscle contractions, and nerve signals [48]. Lastly, there is some evidence that the availability of iron, also deficient in the nutritional imbalance algorithm, in processed or cooked foods may not reflect the availability in the original food [38]. This lack of absorption may contribute, alongside physiological muscle atrophy during aging, to the deterioration of muscle function and strength, as iron is also an essential component of myoglobin and mitochondrial enzymes [50].

### Strengths and limitations

Strengths of this study include its large, population-based design, several anthropometric and dietary variables related to frailty and based on solid statistical algorithms and evidence-based references. Some limitations must be acknowledged. The cross-sectional nature precludes causal inference on findings and, although comprehensive, the dietary database lacked quantification of a small NOVA food category, including butter, olive oil, and seed oil. Also, the FFQ dependence on self-reported information is a limit because of the impossibility of making independent verification. Furthermore, the models were built assuming that the foods classified by NOVA processing category were consumed separately, which would be unrealistic.

## Conclusion

The present study offers further evidence of the food processing contribution to poor nutrition in the aging population and adds to the understanding of nutritional screening milestones. However, our findings mirror a rural Mediterranean population, and so might be underestimated compared to more continental populations on which the association might show an even greater effect.

Monitoring nutrition in aging by taking advantage of new diagnostic algorithms to predict adverse health outcomes may be the best way to target early action. We are confident that greater evidence will help to design better strategies to address dietary concerns. Indeed, a consensus about dietary guidelines for the older population is urgently needed, and the burden of food processing should be taken into account when devising strategies to ease the healthcare burden imposed by the multifaceted frailty syndrome.

### Supplementary information

Below is the link to the electronic supplementary material.

Supplementary file1 (DOCX 21 KB)

## Data Availability

The datasets analyzed during the current study are available from the corresponding author (R.Z.) upon reasonable request.
