# Correction to: Ultra‑processed food consumption and nutritional frailty in older age

**DOI:** 10.1007/s11357-024-01088-1

**Published:** 2024-02-20

**Authors:** Roberta Zupo, Rossella Donghia, Fabio Castellana, Ilaria Bortone, Sara De Nucci, Annamaria Sila, Rossella Tatoli, Luisa Lampignano, Giancarlo Sborgia, Francesco Panza, Madia Lozupone, Giuseppe Colacicco, Maria Lisa Clodoveo, Rodolfo Sardone

**Affiliations:** 1 Unit of Data Sciences and Technology Innovation for Population Health, National Institute of Gastroenterology IRCCS “Saverio de Bellis”, Research Hospital, Castellana Grotte, Bari, Italy; 2 Institute of Clinical Physiology, National Research Council (IFC-CNR), Pisa, Italy; 3 Department of Translational Biomedicine and Neuroscience, University of Bari Aldo Moro, Bari, Italy; 4 Department of Translational Biomedicine and Neuroscience (DiBrain), University of Bari Aldo Moro, Bari, Italy; 5 Department of Interdisciplinary Medicine, University of Bari Aldo Moro, Bari, Italy; 6 Local Healthcare Authority of Taranto, Taranto, Italy

**Correction to: GeroScience (2023) 45:2229–2243**

10.1007/s11357-023-00753-1

The original version of this article unfortunately contained an error in the affiliation section.

The affiliation 1 should be as follows:

Unit of Data Sciences and Technology Innovation for Population Health, National Institute of Gastroenterology IRCCS "Saverio de Bellis", Research Hospital, Castellana Grotte, Bari, Italy

The corrected affiliation 1 is shown below.

