# Supplementary material for: Ultra-processed food consumption and nutritional frailty in older age
Source: GeroScience. 2023 Feb 24;45(4):2229–43. doi: 10.1007/s11357-023-00753-1 (PMC10651811; doi:10.1007/s11357-023-00753-1)
Supplement: Supplementary file 1 — Supplementary file1 (DOCX 21 KB) [file 11357_2023_753_MOESM1_ESM.docx]

**Supplementary Table 1.** Concordance of the single foods in the questionnaire and food grouping according to the NOVA classification.

| Group 1 – Unprocessed or minimally processed foods | Pasta asciutta (*pasta*), Riso o risotti (*rice or risotti*), Pastina o riso in brodo (*pasta or rice in broth*), Vitello (*veal*), Cavallo (*horse*), Maiale (*Pork*), Fegato (*liver*), Agnello (*lamb*), Pollo (*chicken*), Coniglio (*rabbit*), Uova (*eggs),* Sogliola-Orata-Dentice-Spigola-Cernia (*sole, sea bream, snapper, sea bass, grouper*), Merluzzo-Razza-Palombo (*codfish, stingray, dogfish*), Triglia-Cefalo-Sgombro (*goatfish, mullet, mackerel*), Acciughe-Sarde (*anchovies, sardines*), Tonno sott’olio (*tuna in oil*), Polpo-Seppie-Calamari-Gamberi (*octopus, cuttlefish, squid, prawns)*, Cozze-Altri frutti di mare (*mussels, other seafoods*), Latte intero (*whole-fat milk*), Latte scremato – parzialmente scremato (*skimmed and semi-skimmed milk*), Ricotta (*cottage cheese*), Yogurt, Cavoli–Cavolfiori-Cime di Rape-Rape (*cabbage, cauliflower, broccoli, green turnips*), Finocchi- Sedano (*fennels, celery,* Pomodori (*tomatoes*), Zucchine-Melanzane (*zucchini, eggplants*), Peperoni (*peppers*), Carciofi (*artichokes*) Cetrioli-cocomeri (*cucumbers*), Arance-Mandarini-Pompelmi (*oranges, tangerines, grapefruits*), Pesche (*peaches*), Fichi (*figs*), Albicocche (*apricots*), Uva (*grapes*), Anguria (*watermelon*), Melone giallo (*melon*), Mele-Pere (*apples, pears*), Kiwi, Ciliege (cherries), Banane, Acqua (*water*), Caffè (*coffee*), Caffè d’orzo (*barley coffee*). |
| --- | --- |
| Group 2 – Processed foods | Pane (*bread*), Salsiccia fresca (*fresh sausages*), Prosciutto crudo (*raw ham*), Salame (*salami*), Tonno sott’olio (*tuna in oil*), Ricotta (*cottage cheese*), Scamorza-Caciottina fresca-Stracchino-Fontina (semi-seasoned Italian cheese), Bel Paese-Gorgonzola (Italian blue cheese), Provolone-Caciocavallo (seasoned Italian cheese), Grana-Parmigiano, Svizzero (S*wiss cheese*), Pecorino-Vacchino (*goat cheese, cow cheese*), Olive da tavola (*olives*), Frutta sciroppata (*fruit in syrup*) |
| Group 3 - Ultra-processed food | Pizza, Focaccia (a typical Apulian bakery product), Mortadella (a typical Italian cured meat), Prosciutto cotto (ham), Margarina (*margarine*), Formaggino, (*cheese spread*), Succhi di frutta (*fruit juice*), Coca Cola – Aranciata – Chinotto (*coke, orange juice, chinotto*), Caramelle (*sweets*), Cioccolata (*chocolate*), Pasticceria (*pastries*), Biscotti – Paste secche (*cookies, biscuits, macaroons*) |

**Supplementary Table 2.** Proportion of individuals by quintiles of exposure to food consumption according to the NOVA classification by quintiles (very low, low, mild, moderate, and high) subdivided by nutritional frailty phenotype (presence/absence).

| Parameters ^*^ | Nutritional Frailty | | |  |  |
| --- | --- | --- | --- | --- | --- |
|  | No  (*n=1581*) | Yes  (*n=604*) | Effect size ^ψ^  (95% C.I.) | | |
| *Unprocessed and Minimally Processed Foods* |  |  |  | | |
| Very low | 103 (12.47) | 178 (30.85) | 0.18 (0.14 to 0.23) | | |
| Low | 133 (16.10) | 148 (25.65) | 0.09 (0.05 to 0.14) | | |
| Mild | 160 (19.37) | 120 (20.80) | 0.01 (-0.03 to 0.06) | | |
| Moderate | 194 (23.49) | 87 (15.08) | -0.08 (-0.12 to -0.04) | | |
| High | 236 (28.57) | 44 (7.63) | -0.21 (-0.25 to -0.17) | | |
| *Processed Foods* |  |  |  | | |
| Very low | 187 (22.64) | 94 (16.29) | -0.06 (-0.10 to -0.02) | | |
| Low | 197 (23.85) | 84 (14.56) | -0.09 (-0.13 to -0.05) | | |
| Mild | 179 (21.67) | 101 (17.50) | -0.04 (-0.08 to 0.0002) | | |
| Moderate | 159 (19.25) | 122 (21.14) | 0.01 (-0.02 to 0.06) | | |
| High | 104 (12.59) | 176 (30.50) | 0.18 (0.13 to 0.22) | | |
| *Ultra-Processed Foods* |  |  |  | | |
| Very low | 160 (19.37) | 121 (20.97) | 0.02 (-0.03 to 0.06) | | |
| Low | 172 (20.82) | 109 (18.89) | -0.02 (-0.06 to 0.02) | | |
| Mild | 173 (20.94) | 107 (18.54) | -0.02 (-0.07 to 0.02) | | |
| Moderate | 166 (20.10) | 115 (19.93) | -0.002 (-0.04 to 0.04) | | |
| High | 155 (18.77) | 125 (21.66) | 0.03 (-0.01 to 0.07) | | |

^*^ The distribution was divided into quintiles.

^ψ^ Hedges' effect size; (95% C.I.), 95% Confidence Intervals.

**Supplementary Table 3.** Sensitivity Analysis for each model.

| Models | C-Statistic | BIC | AIC | Pseudo-R^2^ |
| --- | --- | --- | --- | --- |
| Univariate |  |  |  |  |
| *Unprocessed and Minimally Processed* | 0.69 | 1768.00 | 1741.77 | 0.09 |
| *Processed Foods* | 0.62 | 1857.45 | 1831.22 | 0.04 |
| *Ultra-Processed Food* | 0.53 | 1933.29 | 1907.06 | 0.002 |
| Adjusted Model 1 |  |  |  |  |
| *Unprocessed and Minimally Processed* | 0.71 | 1759.51 | 1722.79 | 0.10 |
| *Processed Foods* | 0.64 | 1857.40 | 1820.68 | 0.05 |
| *Ultra-Processed Food* | 0.58 | 1925.46 | 1888.74 | 0.01 |
| Adjusted Model 2 |  |  |  |  |
| *Unprocessed and Minimally Processed* | 0.71 | 1766.64 | 1724.68 | 0.10 |
| *Processed Foods* | 0.64 | 1864.33 | 1822.36 | 0.05 |
| *Ultra-Processed Food* | 0.58 | 1932.35 | 1890.38 | 0.01 |
| Adjusted Model 3 |  |  |  |  |
| *Unprocessed and Minimally Processed* | 0.76 | 1714.31 | 1640.86 | 0.15 |
| *Processed Foods* | 0.73 | 1785.15 | 1711.70 | 0.11 |
| *Ultra-Processed Food* | 0.60 | 1959.38 | 1885.94 | 0.02 |

Abbreviations: AIC, Akaike Information Criterion; BIC, Bayesian Information Criterion.
